# Phase 1, pharmacogenomic, dose‐expansion study of pegargiminase plus pemetrexed and cisplatin in patients with ASS1‐deficient non‐squamous non‐small cell lung cancer

**DOI:** 10.1002/cam4.4196

**Published:** 2021-08-12

**Authors:** Peter W. Szlosarek, Akhila G. Wimalasingham, Melissa M. Phillips, Peter E. Hall, Pui Ying Chan, John Conibear, Louise Lim, Sukaina Rashid, Jeremy Steele, Paula Wells, Chiung‐Fang Shiu, Chih‐Ling Kuo, Xiaoxing Feng, Amanda Johnston, John Bomalaski, Stephen Ellis, Marianne Grantham, Michael Sheaff

**Affiliations:** ^1^ Center for Cancer Biomarkers and Biotherapeutics Barts Cancer Institute (BCI) – A Cancer Research UK Center of Excellence Queen Mary University of London John Vane Science Center London UK; ^2^ Department of Medical Oncology Barts Health NHS Trust St Bartholomew’s Hospital London UK; ^3^ Department of Clinical Oncology Barts Health NHS Trust St Bartholomew’s Hospital London UK; ^4^ Polaris Pharmaceuticals, Inc. San Diego California USA; ^5^ Department of Diagnostic Imaging Barts Health NHS Trust St Bartholomew’s Hospital London UK; ^6^ Cytogenetics and Molecular Haematology, Pathology and Pharmacy Building Barts Health NHS Trust Royal London Hospital London UK; ^7^ Department of Histopathology, Pathology and Pharmacy Building Barts Health NHS Trust Royal London Hospital London UK

**Keywords:** ADIPemCis, arginine, arginine deiminase, ASS1, KRAS, non‐squamous NSCLC, p53, PD‐L1

## Abstract

**Introduction:**

We evaluated the arginine‐depleting enzyme pegargiminase (ADI‐PEG20; ADI) with pemetrexed (Pem) and cisplatin (Cis) (ADIPemCis) in ASS1‐deficient non‐squamous non‐small cell lung cancer (NSCLC) via a phase 1 dose‐expansion trial with exploratory biomarker analysis.

**Methods:**

Sixty‐seven chemonaïve patients with advanced non‐squamous NSCLC were screened, enrolling 21 ASS1‐deficient subjects from March 2015 to July 2017 onto weekly pegargiminase (36 mg/m^2^) with Pem (500 mg/m^2^) and Cis (75 mg/m^2^), every 3 weeks (four cycles maximum), with maintenance Pem or pegargiminase. Safety, pharmacodynamics, immunogenicity, and efficacy were determined; molecular biomarkers were annotated by next‐generation sequencing and PD‐L1 immunohistochemistry.

**Results:**

ADIPemCis was well‐tolerated. Plasma arginine and citrulline were differentially modulated; pegargiminase antibodies plateaued by week 10. The disease control rate was 85.7% (*n* = 18/21; 95% CI 63.7%–97%), with a partial response rate of 47.6% (*n* = 10/21; 95% CI 25.7%–70.2%). The median progression‐free and overall survivals were 4.2 (95% CI 2.9–4.8) and 7.2 (95% CI 5.1–18.4) months, respectively. Two PD‐L1‐expressing (≥1%) patients are alive following subsequent pembrolizumab immunotherapy (9.5%). Tumoral ASS1 deficiency enriched for p53 (64.7%) mutations, and numerically worse median overall survival as compared to ASS1‐proficient disease (10.2 months; *n* = 29). There was no apparent increase in KRAS mutations (35.3%) and PD‐L1 (<1%) expression (55.6%). Re‐expression of tumoral ASS1 was detected in one patient at progression (*n* = 1/3).

**Conclusions:**

ADIPemCis was safe and highly active in patients with ASS1‐deficient non‐squamous NSCLC, however, survival was poor overall. ASS1 loss was co‐associated with p53 mutations. Therapies incorporating pegargiminase merit further evaluation in ASS1‐deficient and treatment‐refractory NSCLC.

## Graphical abstract

We evaluated the arginine‐depleting enzyme pegargiminase (ADI‐PEG20; ADI) with pemetrexed (Pem) and cisplatin (Cis) (ADIPemCis) in ASS1‐deficient non‐squamous non‐small cell lung cancer (NSCLC) via a phase 1 dose‐expansion trial with exploratory biomarker analysis. ADIPemCis was safe and highly active in patients with ASS1‐deficient non‐squamous NSCLC, however, survival was poor overall. ASS1 loss was co‐associated with p53 mutations providing a developmental pathway for further testing pegargiminase in ASS1‐deficient and treatment‐refractory NSCLC.

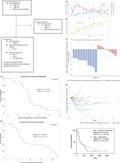

## 1 INTRODUCTION

Globally, lung cancer is the leading cause of cancer‐related mortality, accounting for almost one in five deaths.^1^ Around 85% of lung cancer patients have non‐small cell lung carcinoma (NSCLC), comprising mostly of adenocarcinoma (40%), squamous carcinoma (30%), and large‐cell undifferentiated carcinoma (10%–15%).^2^ Standard of care for patients with first‐line metastatic non‐squamous NSCLC––in the absence of an oncogenic driver such as an EGFR mutation or ALK translocation––is immune checkpoint blockade either alone or in combination with platinum‐based chemotherapy (pemetrexed or paclitaxel‐bevacizumab). While the 5‐year survival has improved from less than 5% a decade ago to over 25% in high (≥50%) PD‐L1‐expressing NSCLC, prognosis for many patients remains poor, emphasizing the need for more options that exploit novel biological pathways.^3^

Arginine is a versatile amino acid involved in the biosynthesis of proteins and regulation of numerous cellular processes, including proliferation by modulating polyamine and nucleotide synthesis, hormone synthesis, cell signaling, and vasodilation via nitric oxide.^4^ Arginine also has a crucial role in immune system regulation.^5^ Normal cells synthesize arginine de novo from citrulline and aspartate via ATP in the urea cycle, however this is dysregulated in arginine auxotrophic cancers––exemplified by loss of the tumor suppressor argininosuccinate synthetase 1 (ASS1)––rendering arginine essential for tumor growth (“arginine auxotrophy”) and thereby leveraging arginine deprivation as an attractive therapeutic strategy.^6^, ^7^ Moreover, arginine deprivation with pegylated arginine deiminase (ADI‐PEG20; ADI; pegargiminase) disrupts thymidine pools and potentiates antifolate cytotoxicity in preclinical ASS1‐deficient tumor models.^8^

In addition to its role as a predictive biomarker for arginine deprivation therapy, ASS1 deficiency is increasingly recognized as a prognostic biomarker for poor survival outcomes in several cancer types, including mesothelioma, sarcomas, bladder, ovarian, and breast cancer by driving increased tumor cell proliferation, invasiveness, and metastasis.^8^, ^9^, ^10^, ^11^, ^12^, ^13^, ^14^ Moreover, based on data from The Cancer Genome Atlas (TCGA), ASS1 loss confers significantly reduced 5‐year survival rates (25% “low” vs. 44% “high” ASS1 expression; *p* = 0.007; *n* = 500) for all stages of lung adenocarcinoma, including patients in adjuvant and metastatic treatment settings.^15^ In particular, low ASS1 combined with high citrin (mitochondrial aspartate transporter) expression conferred poor survival outcomes in both adenocarcinoma and squamous cell lung cancer consistent with the redirection of aspartate for enhanced pyrimidine synthesis in urea cycle‐deficient cancers.^16^ Although preclinically ASS1 loss is linked to chemorefractoriness, specific data on the impact of systemic therapy in ASS1 low and high subsets in NSCLC are not currently available.

In the phase I dose‐escalation TRAP study^17^ we showed that ADI‐PEG20 with pemetrexed (Pem) and cisplatin (Cis) chemotherapy (ADIPemCis) was tolerable and produced a 100% disease control rate (DCR) and an objective response rate of 78% in nine patients with ASS1‐deficient thoracic cancers (non‐squamous NSCLC = 4; mesothelioma = 5) (Beddowes et al., 2017). However, while the median overall survival––prior to the approval of immune checkpoint therapy––was promising at 13.9 months, the small patient thoracic cohort precluded any firm survival estimates. Recently, we have reported on the safety and activity of ADIPemCis in dose‐expansion cohorts in patients with high‐grade glioma (*n* = 10), malignant pleural mesothelioma (*n* = 31), and a placebo‐controlled phase 3 trial of ADIPemCis is now underway in non‐epithelioid mesothelioma based on a robust survival signal.^17^, ^18^

Therefore, we tested the hypothesis that targeting essential arginine would benefit patients with non‐squamous NSCLC characterized by loss of the ASS1 tumor suppressor. We treated a dose‐expansion cohort of 21 patients with ASS1‐deficient non‐squamous non‐small cell lung cancer at the recommended phase 2 dose (RP2D) of ADI‐PEG 20 (36 mg/m^2^) in combination with standard doses of pemetrexed and cisplatin. The main aims of this phase 1 dose‐expansion study were to define further the safety, preliminary activity, and survival estimates of the ADIPemCis triplet in patients with non‐squamous NSCLC. Additionally, we sought to translate the correlative molecular biomarkers of ASS1 deficiency in non‐squamous NSCLC by next‐generation sequencing, characterize the levels of PD‐L1 expression immunohistochemically, and explore the treatment resistance.

## 2 PATIENTS AND METHODS

### 2.1 Patient eligibility

Patients were aged 18 years or over, with histologically proven argininosuccinate synthetase 1 (ASS1)‐deficient stage IIIB or IV non‐squamous NSCLC (v. Beddowes et al. for methods). Patients had evaluable disease by Response Evaluation Criteria in Solid Tumors criteria (RECIST) 1.1. All patients were chemotherapy naïve. Subjects with EGFR or ALK mutation must have had an EGFR tyrosine kinase inhibitor (TKI) or ALK inhibitor, and must have progressed or been shown to be intolerant of this therapy prior to enrolling into the study. Additional criteria included Eastern Cooperative Oncology Group performance status 0 or 1, adequate hematologic, renal and hepatic function, and a minimum of 12‐week life expectancy. Exclusion criteria included symptomatic brain or spinal cord metastases, recent major surgery, significant concomitant illness, allergy to platinum salts, or pegylated agents. The protocol amendment for the dose‐expansion cohort specified the enrollment of up to 30 patients with non‐squamous NSCLC at the RP2D and required written informed signed consent.

### 2.2 Study design and treatment

This was a dose‐expansion cohort phase I study in up to 30 patients with non‐squamous NSCLC testing the recommended phase II dose (RP2D) of 36 mg/m^2^ weekly intramuscular (IM) ADI‐PEG 20 plus 75 mg/m2 cisplatin and 500 mg/m2 pemetrexed given every 3 weeks, and derived from the prior dose‐escalation TRAP study.^19^ The initial dose of ADI‐PEG20 was given 48 h prior to the first dose of intravenous chemotherapy. Standard premedication to reduce pemetrexed toxicity was given, namely dexamethasone, daily folic acid (400 µg), and IM hydroxycobalamin (1000 µg) every 9 weeks, starting at least 7 days prior to first dose. Patients received a maximum of four cycles (12 weeks) after which patients who had stable disease or partial response were eligible to continue maintenance single‐agent ADI‐PEG20 or pemetrexed until disease progression or withdrawal. Blood samples were taken at baseline, prior to each cycle of ADIPemCis and on progression of disease or study withdrawal. Baseline tumor biopsies were mandatory and were optional at disease progression.

### 2.3 Study endpoints

The data lock was performed on the 18 June 2020. The primary endpoints of the dose‐expansion phase were to assess the safety and tolerability of ADIPemCis using National Cancer Institute Common Terminology Criteria for Adverse Events (CTCAE) Version 4.03; and to estimate the preliminary efficacy or response rate (RR) using RECIST 1.1, by computed tomography (CT) every 6 weeks during ADIPemCis therapy and then every 8 weeks during pegargiminase or pemetrexed maintenance therapy. Secondary endpoints were to calculate the median progression‐free survival (PFS), median overall survival (OS), pharmacodynamics, and immunogenicity. For the translational endpoints, next‐generation sequencing was performed on baseline tumor DNA examining the different somatic variants detected using Ion AmpliSeq Cancer Hotspot Panel v2 (Thermo Fisher Scientific). PD‐L1 expression was assessed using the 22C3 immunohistochemistry (IHC) assay and reported according to the standard criteria (>50%; 1%–49%; <1% expression). Rebiopsied tumor tissue at relapse was assessed for ASS1 IHC using the monoclonal antibody 195‐21‐1 from Polaris Pharmaceuticals, Inc.

### 2.4 Statistical analyses

No formal sample size calculation was made for the dose‐expansion TRAP study in patients with non‐squamous NSCLC, which aimed to recruit up to 30 patients as per protocol. AEs were collated, and response rates, PFS, and OS were graphed using Prism 8 Version 8.4.3. This trial is registered with clinicaltrials.gov, number.^33^

## 3 RESULTS

### 3.1 Patient demographics

**FIGURE 1:**
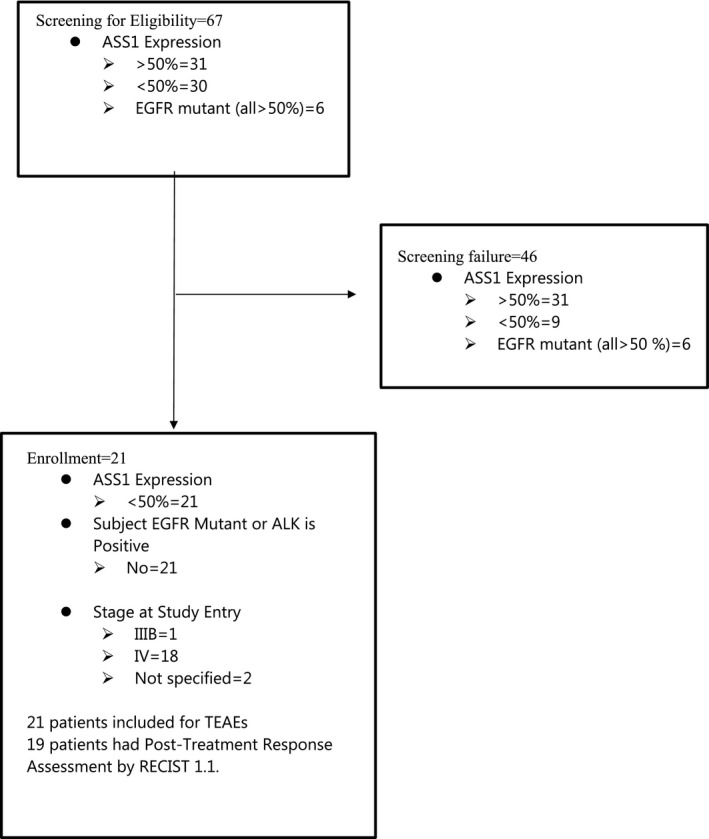
CONSORT diagram

Patient enrollment into the dose‐expansion study began in March 2015 and was completed in July 2017. Sixty‐seven patients with non‐squamous NSCLC were screened, and 21 patients with ASS1‐deficient tumors were enrolled for the treatment with ADIPemCis as summarized in Figure 1. None of the patients with ASS1‐deficient disease had EGFR mutations or ALK rearrangements. The majority had stage IV lung adenocarcinoma (LUAC) which included patients with initial presentations of superior vena cava obstruction (*n* = 1), pericardial tamponade (*n* = 1), and multiple brain metastases (*n* = 1) requiring stabilization prior to study entry (*n* = 18/21; 85.7%). All subjects were included for the safety, while 19 were evaluable by RECIST 1.1 (Table 1). Patients with ASS1‐proficient non‐squamous NSCLC who received first‐line platinum and pemetrexed chemotherapy were followed up for survival only.

**TABLE 1:** Demographics of patients receiving ADIPemCis

Characteristic	Number of patients (n = 21)
Age, years	
Median	60
Range	39–78
Gender	
Male	13
Female	8
ECOG performance status	
0	6
1	15
Prior therapy	
Surgery	5
External beam radiotherapy	3
Disease stage^a^	
IIIB	1
IV	18
Unknown	2
Histology	
Adenocarcinoma	19
Large cell carcinoma	1
Pleomorphic (Giant cell) carcinoma	1

Abbreviation: ECOG, Eastern Cooperative Oncology Group.

^a^ AJCC eighth edition TNM staging for lung cancer.

### 3.2 Safety

Consistent with the prior dose‐escalation study, ADIPemCis treatment was well‐tolerated. Treatment‐emergent adverse events were reported in all 21 patients: the majority were related to cisplatin and pemetrexed (81%; *n* = 17/21), and the rest to pegargiminase (52.4%; *n* = 11/21). Grade 1/2 adverse events occurred in 36.4% (*n* = 8/21) and were mostly due to nausea, anorexia, and fatigue, while Grade 3 was reported in 47.6% (*n* = 10/21) patients; adverse events of Grade 4 occurred in 4.8% (*n* = 1/21) and grade 5 in 9.5% (*n* = 2/21) patients, the latter due to disease progression (Table 2; Table S1).

**TABLE 2:** Overall adverse events

Overall AE^a^ summary	Number of subject (*n* = 21)	**%**
Total number of adverse event (AE)	197	
Number of subject reporting at least one AE	21	100.0
AE by severity		
Grade 1	3	14.3
Grade 2	5	23.8
Grade 3	10	47.6
Grade 4	1	4.8
Grade 5 (peritoneal PD; CNS PD)	2	9.5
Number of subject reporting at least one SAE	12	57.1
AE related to ADI‐PEG 20	11	52.4
AE related to cisplatin	17	81.0
AE related to pemetrexed	17	81.0

Abbreviations: CNS, central nervous system; PD, progressive disease.

^a^ AE is defined as any untoward medical occurrence in a subject administered with ADIPemCis and that does not necessarily have a causal relationship with the treatment.

### 3.3 Pharmacodynamics

**FIGURE 2:**
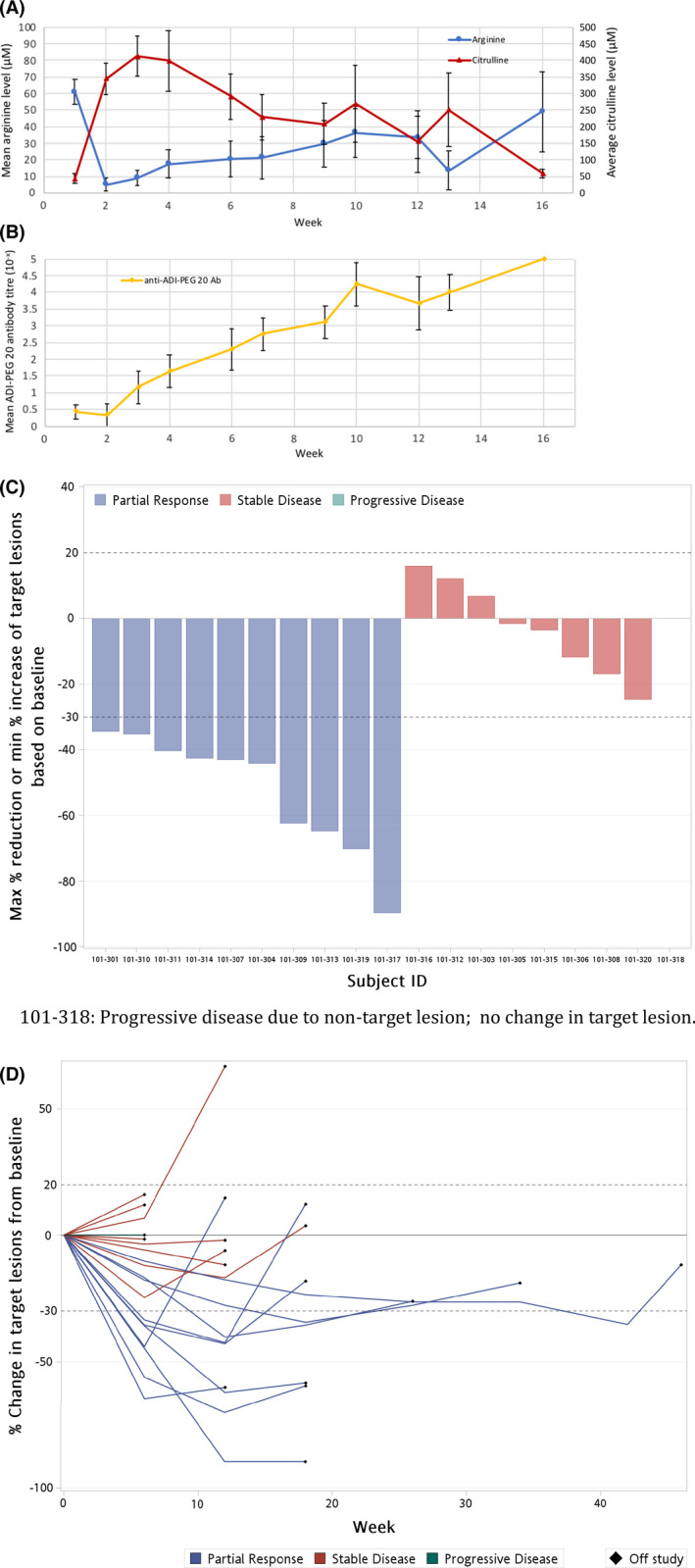
Pharmacodynamics and response. (A) Pharmacodynamics of arginine and citrulline in patients treated with ADIPemCis. Serum [arginine] and [citrulline] are shown by week of treatment (means ± SEM). (B) Serum levels of anti‐ADI‐PEG 20 antibodies in all patients by week of ADIPemCis (Mean ± SEM); Ab, Antibody. (C) Waterfall plot of response by RECIST 1.1. to ADIPemCis. (D) Spider plots showing response duration to ADIPemCis

Pegargiminase decreased plasma arginine with a reciprocal increase in plasma citrulline levels in patients and is shown in Figure 2A. Plasma levels of the amino acids remained differentially altered compared with pre‐treatment levels, however, recovery to pre‐treatment levels was evident with the fewer number of on‐treatment patients by 16 weeks. There was a fourfold increase in the titer of ADI‐PEG20 antibodies by 16 weeks consistent with previous studies and is shown in Figure 2B.

### 3.4 Efficacy

**FIGURE 3:**
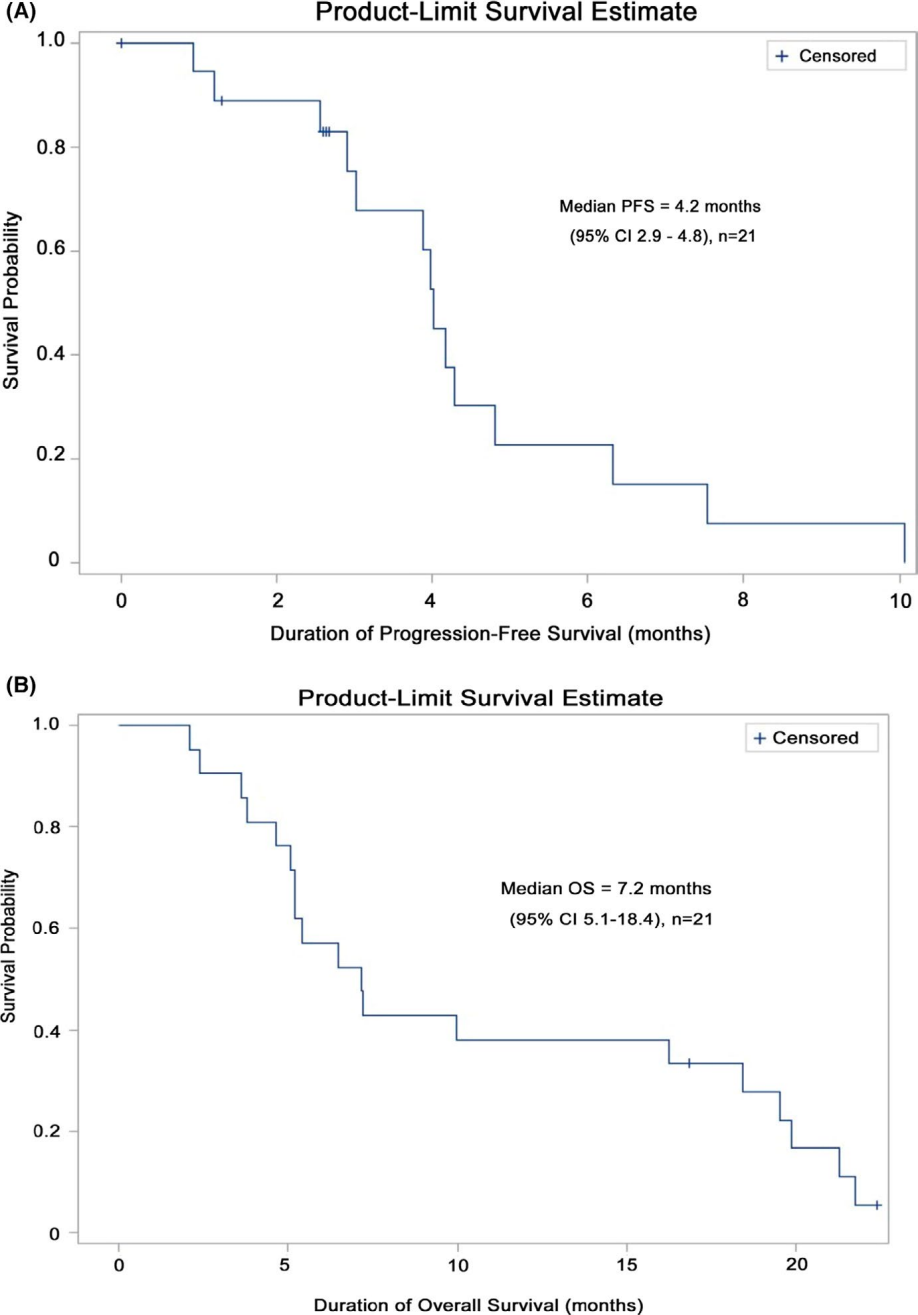
Survival outcomes for ASS1‐deficient patients (A) Progression‐free survival. (B) Kaplan–Meier survival estimates

ADIPemCis treatment induced a disease control rate of 85.7% (*n* = 18/21; 95% CI 63.7%–97%), and a partial response rate of 47.6% (*n* = 10/21, 95% CI 25.7%–70.2%; or *n* = 10/18 or 55.6% of evaluable patients), in a cohort of patients with non‐squamous NSCLC enriched by ASS1 loss, summarized in Figure 2C,D. The median PFS and OS were 4.2 (95% CI 2.9–4.8) and 7.2 (95% CI 5.1–18.4) months, respectively, and are shown in Figure 3.

### 3.5 Molecular biomarkers

To understand the disconnect between the high partial response rate and the unexpectedly short median PFS and OS, NGS was performed on the baseline tumor biopsies. This revealed a wide spectrum of p53 mutations in 64.7% of patients and KRAS mutations in 35.3% of patients (G12C, G13C, G12D, and Q61H) in ASS1‐deficient non‐squamous NSCLC. Additional mutations were identified including in HRAS, CDKN2A, BRAF (V600E), ATM, RB1, and APC (Table 3). Overall, the expression of PD‐L1 ≥1% was reported in 44.4% of patients (*n* = 8/18), while 55.6% of patients (*n* = 10/18) were PD‐L1 negative by the 22C3 IHC assay. Patients with tumoral PD‐L1 ≥1% expression and disease progression were eligible for second‐line pembrolizumab; two patients are in remission at 40 and 47 months from treatment with ADIPemCis following 2 years of immunotherapy (9.5%). Finally, to study drug resistance, three patients receiving maintenance ADI‐PEG20 consented to a rebiopsy revealing ASS1 re‐expression in one patient (*n* = 1/3; 33%).

**TABLE 3:** Individual patient and tumor characteristics

Subject	Age	Gender	Diagnosis	Disease presentation	PD‐L1 expression^a^	NGS somatic variants^b^		
						*TP53*	*KRAS*	Other
101‐301	64	M	ADC	Lung, LN	<1%	WT	G12C	
101‐302	54	M	ADC	LN, Adrenal	>50%	R158L	WT	
101‐303	51	F	ADC	Lung	N/A	N/A	N/A	
101‐304	60	M	ADC	Lung	3%	G245V	G12C	
101‐305	45	M	Large cell	Lung, LN, Peritoneal	<1%	G334V	WT	
101‐306	58	M	ADC	Lung, LN	N/A	WT	WT	
101‐307	56	F	ADC	Lung, LN, Bone	<1%	WT	G12D	
101‐308	68	M	ADC	LN, Bone, Muscle	<1%	R248Q	WT	*ATM*
101‐309	63	M	ADC	Lung, LN	<1%	N/A	N/A	
101‐310	63	F	ADC	LN, Bone	5%–10%	G199V	WT	*BRAF* (V600E)
101‐311	39	M	ADC	Lung, LN, Liver	<1%	N/A	N/A	
101‐312	62	M	ADC	Lung, Adrenal, Bone	<1%	WT	Q61H	
101‐313	60	F	ADC	Lung, LN, Pleural, Pericardial Bone	80%–90%	E285V	G13C	
101‐314	50	M	ADC	Lung, LN, Liver, Pleural	<1%	WT	WT	
101‐315	65	M	Giant cell	Lung, LN, Adrenal	100%	Y126D	WT	
101‐316	60	F	ADC	Lung, LN, Adrenal	<1%	WT	G12D	*APC*
101‐317	59	F	ADC	Lung, LN, Liver, Bone	<1%	G244R	WT	*RB1; ERBB2*
101‐318	53	M	ADC	Lung	10%–20%	G245V	WT	*CDKN2A; HRAS*
101‐319	70	M	ADC	Lung, LN	60%–70%	R248L	WT	
101‐320	78	M	ADC	Lung, LN	60%–70%	R249T	WT	*JAK3* P132T
103‐301	61	F	ADC	N/A	N/A	N/A	N/A	

Abbreviations: ADC, adenocarcinoma; F, female; M, male; N/A, not available; NGS, next‐generation sequencing; PD‐L1, programmed cell death‐ligand 1.

^a^ PD‐L1 expression assessed using the 22C3 immunohistochemistry assay.

^b^ Somatic variants detected using Ion AmpliSeq^TM^ Cancer Hotspot Panel v2 analysis of tumor DNA.

### 3.6 ASS1‐positive group

**FIGURE 4:**
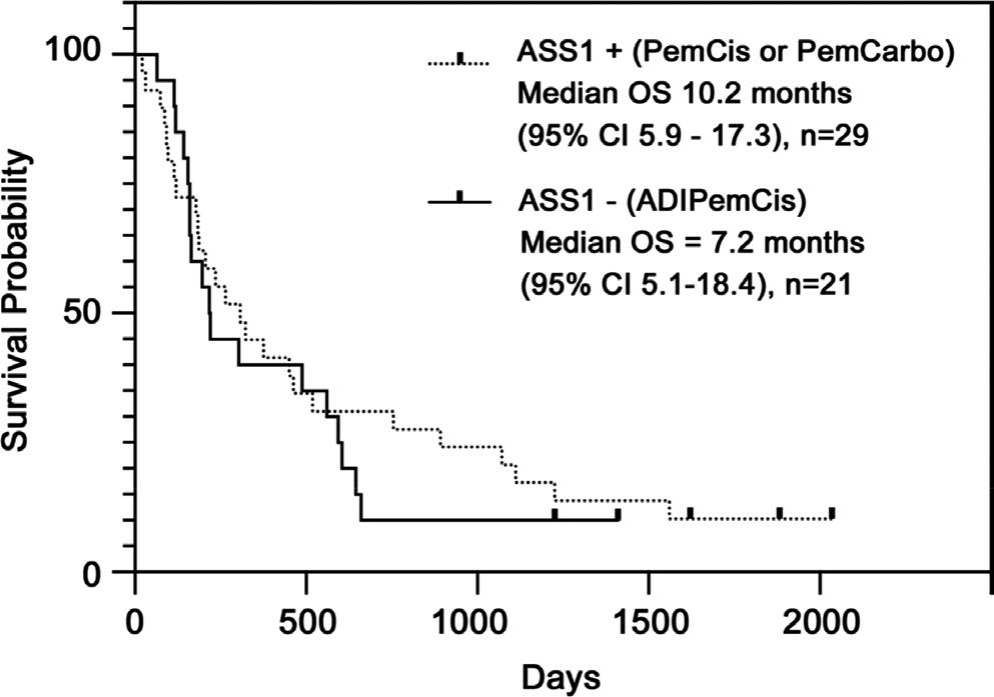
Survival outcomes for ASS1‐proficient patients Kaplan–Meier survival estimates for ASS1‐proficient patients screened as part of this study (*n* = 29) and treated with standard of care cisplatin and pemetrexed chemotherapy and compared to ASS1‐deficient patients receiving ADIPemCis (*n* = 21)

Retrospectively, we determined the median OS of patients with ASS1‐proficient non‐squamous NSCLC screened as part of this study (*n* = 35). Following exclusion of six ASS1‐positive patients with EGFR mutations, who all received first‐line therapy with a tyrosine kinase inhibitor (TKI: gefitinib, erlotinib, or afatinib), the median OS for patients receiving first‐line platinum (cisplatin or carboplatin) and pemetrexed chemotherapy was 10.2 months (*n* = 29; 95% CI 5.9–17.3) and is depicted in Figure 4. This included two patients with ALK rearrangements who were eligible for second‐line crizotinib. Overall, three patients are alive from the ASS1‐proficient group including a patient with ALK‐positive disease on crizotinib (10.3%).

## 4 DISCUSSION

This expansion study of ADIPemCis at the RP2D confirmed good safety and a high rate of disease control and partial response (~50%) in patients with ASS1‐deficient non‐squamous NSCLC consistent with the dose‐escalation study.^19^ However, the encouraging response rate did not translate into a prolonged median OS (7.2 months) when contrasted with ASS1‐proficient disease (10.2 months) or compared with ASS1‐agnostic historical controls (10.4–12.6 months) treated with platinum and pemetrexed alone.^20^ Notably, p53 and KRAS mutations were co‐associated with ASS1‐deficient non‐squamous NSCLC contributing to the poor clinical outcomes; PD‐L1 expression was commonly absent (<1% staining by the 22C3 assay). In contrast, EGFR‐mutant and ALK rearranged non‐squamous NSCLC tumors were all ASS1‐expressors for which TKI therapies have revolutionized patient management.

Since completing the ADIPemCis dose‐expansion cohort, chemoimmunotherapy with PD1 and PD‐L1 inhibitors has overtaken platinum‐doublet chemotherapy reaching a median OS of 22 months for pembrolizumab with platinum–pemetrexed (vs. 10.7 months for platinum and pemetrexed) in the KEYNOTE‐189 study, and 19.2 months for atezolizumab with bevacizumab, carboplatin, and paclitaxel (vs. 14.7 months for bevacizumab, carboplatin, and paclitaxel) in the IMpower150 study.^21^, ^22^ Further optimization of chemoimmunotherapy especially in PD‐L1‐negative expressors is a priority area, as benefits are modest compared with disease exhibiting high (≥50%) PD‐L1 expression, where single‐agent PD1i/PDL1i immunotherapy is an approved standard of care.^23^, ^24^ Two patients treated with ADIPemCis with ≥1% tumoral PDL1 expression remain alive and in remission, post‐pembrolizumab immunotherapy. Both harbored mutations in either JAK3 or CDKN2A which are known to upregulate cancer immune responses in NSCLC.^25^, ^26^

PD‐L1 <1% expression was reported in 55.6% of patients enrolled on ADIPemCis, a prevalence that is significantly higher than that reported across key studies of pembrolizumab in NSCLC (33%), but consistent with real‐world data (48%–56.4%).^27^, ^28^, ^29^ Recent work suggests that arginine deprivation may potentiate immune checkpoint inhibition and turn “cold” tumors “hot” by increasing the PD‐L1 expression and T‐cell infiltration.^30^, ^31^ Moreover, urea cycle dysregulated cancers are differentially sensitive to immune checkpoint therapies and further clinical testing will be needed to validate these preclinical data.^32^ Specifically, a phase 1 study of pembrolizumab and pegargiminase has completed accrual and, while patients with NSCLC were not enrolled, this immunometabolic approach was tolerable with partial responses in 24% of subjects.^33^

The 41% higher prevalence of p53 mutations in our study compared to that expected for patients with LUAC (i.e., 64.7% vs. 46%) is intriguing.^34^ Miyamoto et al. showed that wild‐type p53 activates ASS1 in response to genotoxic and nutrient stress driving arginine biosynthesis in line with its known homeostatic functions as a key tumor suppressor pathway.^35^, ^36^ Specifically, transfection of wild‐type p53 in p53‐deficient (NSCLC) or p53‐mutant (astrocytoma) cancer cell lines was sufficient to restore ASS1 expression. Moreover, the p53‐mediated regulation of ASS1 was cell type‐specific, consistent with the diverse roles of arginine in cellular homeostasis. Thus, our study links various p53 mutations to deregulation of ASS1 in clinical samples for the first time, and also provides an explanation for the poor overall survival seen in the non‐squamous NSCLC patient cohort. Multiple studies have identified p53 mutations as critical in lung carcinogenesis and more recently as a key biomarker of adverse outcomes in NSCLC.^37^, ^38^, ^39^, ^40^ Further analysis of the relationship between p53 and ASS1 is warranted, particularly in squamous NSCLC where p53 mutations occur with the highest frequency (81%). Preclinically, ADI‐PEG20 combined with a taxane potentiates gemcitabine cytotoxicity, and is an approach that may have utility in patients with squamous NSCLC.^41^ Interestingly, our NSCLC cohort data validate studies showing high rates of ASS1 loss in non‐epithelioid mesothelioma, where p53 mutations have been identified to the exclusion of epithelioid mesothelioma, and underlie similar poor outcomes to platinum–pemetrexed chemotherapy.^42^, ^43^

Mutations in KRAS were the second most common finding by NGS in our patient cohort with a modestly higher prevalence compared to patients with unselected LUAC reported in the recent published AACR Project GENIE, namely 35.3% versus 26.7%.^44^ However, KRAS mutations have been described with a frequency of 33% in the TCGA dataset for LUAC thus a molecular link with ASS1 requires additional scrutiny.^45^ Two patients in our study achieved PRs on ADIPemCis despite co‐mutations of p53 and KRAS mutations which portend a poor survival with disease refractory to chemotherapy and radiotherapy.^46^, ^47^ None of the patients displayed STK11/LKB1 mutations which, linked to KRAS mutations, yield a median OS of 6.4 months versus 16 months for KRAS mutations alone.^48^ Interestingly, KEAP1 mutations, which were not represented in the NGS panel, are frequently co‐mutated with LKB1 and similarly herald a poor prognosis characterized by glutamine addiction and sensitivity to glutaminase inhibition in KRAS‐mutant LUAC.^49^ Site‐specific differences have also been reported in the median OS for patients with KRAS mutations and LUAC: 3.7 months for bone metastases versus 9.7 months without bone involvement.^50^ Importantly, the recent development of K12C‐targeted drugs will leverage novel opportunities for patients with KRAS co‐mutated tumors.^51^ Preclinically, we have identified enhanced disease control of an ASS1‐deficient and K12D KRAS‐mutant NSCLC cell line sensitive to ADI‐PEG20 and PD1 blockade.^52^

As noted earlier, several studies reinforce that ASS1 deficiency is a poor prognostic biomarker in a variety of solid malignancies.^8^, ^9^, ^10^, ^11^, ^12^, ^13^, ^14^, ^16^ In the present study, the median OS for ASS1‐deficient compared to ASS1‐proficent patients was numerically shorter by 3 months (i.e., 7.2 vs. 10.2 months) but, based on our small patient population, was not significant. While our study implicate p53, and, in part, KRAS mutations, in the overall poor prognosis, the rapid emergence of resistance to pegargiminase requires greater scrutiny. Thus, several mechanisms are apparent, namely, tumoral re‐expression of ASS1, stromal support via infiltrating macrophages identified in the earlier mesothelioma TRAP dose‐expansion cohort, and potentially drug‐dependent resistance, evidenced by an increasing titer of pegargiminase antibodies and a concomitant rise in arginine levels by week 16.^17^ While the pharmacodynamic response was less durable––possibly due to low numbers of patients sampled by week 16––compared with the dose‐escalation ADIPemCis study, it was nonetheless more robust than that reported for doublet chemotherapy with ADI‐PEG20 and docetaxel.^19^, ^53^ Lastly, preclinical studies in NSCLC also support autophagy as a key modulator of KRAS‐dependent growth warranting further clinical study.^54^

In summary, ADIPemCis is safe and active in an expansion cohort of patients with ASS1‐deficient non‐squamous NSCLC. Our data reveal that tumoral ASS1 deficiency enriches for a poor prognosis group of patients characterized by frequent p53 mutations, in which arginine deprivation may provide additional therapeutic benefit warranting further investigation with rationally selected agents.

## ETHICAL CONSIDERATIONS

The clinical protocol (ClinicalTrials.gov identifier NCT02029690) was approved by Leeds East Research Ethics Committee (14/YH/0090) and was sponsored by Polaris Pharmaceuticals, Inc.

## CONFLICT OF INTEREST

PW Szlosarek received support from the Higher Education Funding Council for England (HEFCE) and research grant support from Polaris Group. Ms Shiu, Ms Kuo, Dr Bomalaski, Dr Johnson, and Dr Feng are paid employees of Polaris Pharmaceuticals. The remaining authors did not report any relevant conflict of interest.

## Supporting information

Table S1

## Data Availability

The data that support the findings of this study are available upon request from the corresponding author. The data are not publicly available due to privacy or ethical restrictions.
