# Supplementary material for: Phase 1, pharmacogenomic, dose‐expansion study of pegargiminase plus pemetrexed and cisplatin in patients with ASS1‐deficient non‐squamous non‐small cell lung cancer
Source: Cancer Med. 2021 Aug 12;10(19):6642–52. doi: 10.1002/cam4.4196 (PMC8495293; doi:10.1002/cam4.4196)
Supplement: Supplementary file 1 — Table S1 [file CAM4-10-6642-s001.docx]

| Grade 1-5 AEs in patients with >=7.5% grade 1-2 events | CTCAE grade 1-2 | CTCAE grade 3 | CTCAE grade 4 | CTCAE grade 5 |
| --- | --- | --- | --- | --- |
| Nausea | 9( 42.9%) | 0 | 0 | 0 |
| Fatigue | 8( 38.1%) | 1( 4.8%) | 0 | 0 |
| Decreased appetite | 7( 33.3%) | 0 | 0 | 0 |
| Pyrexia | 6( 28.6%) | 0 | 0 | 0 |
| Constipation | 5( 23.8%) | 0 | 0 | 0 |
| Headache | 5( 23.8%) | 0 | 0 | 0 |
| Haemoptysis | 4( 19.0%) | 0 | 0 | 0 |
| Stomatitis | 4( 19.0%) | 1( 4.8%) | 0 | 0 |
| Vomiting | 4( 19.0%) | 0 | 0 | 0 |
| Arthralgia | 3( 14.3%) | 1( 4.8%) | 0 | 0 |
| Cough | 3( 14.3%) | 0 | 0 | 0 |
| Dyspnoea exertional | 3( 14.3%) | 0 | 0 | 0 |
| Increased upper airway secretion | 3( 14.3%) | 0 | 0 | 0 |
| Neuropathy peripheral | 3( 14.3%) | 0 | 0 | 0 |
| Anaemia | 2( 9.5%) | 0 | 0 | 0 |
| Chest pain | 2( 9.5%) | 0 | 0 | 0 |
| Dysgeusia | 2( 9.5%) | 0 | 0 | 0 |
| Dyspepsia | 2( 9.5%) | 0 | 0 | 0 |
| Dyspnoea | 2( 9.5%) | 0 | 0 | 0 |
| Gastrooesophageal reflux disease | 2( 9.5%) | 0 | 0 | 0 |
| Insomnia | 2( 9.5%) | 0 | 0 | 0 |
| Nasal discomfort | 2( 9.5%) | 0 | 0 | 0 |
| Oral candidiasis | 2( 9.5%) | 0 | 0 | 0 |
| Rash | 2( 9.5%) | 0 | 0 | 0 |
